# Na^+^/K^+^ Balance and Transport Regulatory Mechanisms in Weedy and Cultivated Rice (*Oryza sativa* L.) Under Salt Stress

**DOI:** 10.1186/s12870-018-1586-9

**Published:** 2018-12-29

**Authors:** Yuhua Zhang, Jiapeng Fang, Xibao Wu, Liyao Dong

**Affiliations:** 1 College of Plant Protection, Nanjing Agricultural University, Nanjing, 210095 China; 2 Key Laboratory of Integrated Pest Management on Crops in East China, Ministry of Agriculture, Nanjing Agricultural University, Nanjing, 210095 China

**Keywords:** NaCl stress, Weedy rice, Na^+^/K^+^ homeostasis, *HKT* family, *NHX* family, Vacuolar *SOS1*

## Abstract

**Background:**

Salinization is a primary abiotic stress constraining global plant growth and production. Weedy rice, though highly homologous to cultivated rice, is more salt tolerant during seed germination and seedling growth; we hypothesize that this is owing to ionic homeostasis and changes in the expression of genes encoding ion transport regulators.

**Results:**

The four different genotypes of weedy (*JYGY-1* and *JYFN-4*) and cultivated (*Nipponbare* and *9311*) rice have different salt-tolerance during seed germination and seedling vegetative growth under salt stress. In this study, Na^+^ and Ca^2+^content increased in weedy and cultivated rice genotypes under salt stress while K^+^ and Mg^2+^decreased; however, *JYGY-1* had the lowest Na^+^/K^+^ ratio of assessed genotypes. Genes in the high-affinity K^+^ transporter (*HKT*) and tonoplast sodium-hydrogen exchanger (*NHX*) families, and salt overly sensitive 1 (*OsSOS1*) have more than 98% homology in amino acid sequences between weedy and cultivated rice genotypes. Under salt stress, the *HKT* family members were differentially expressed in the roots and shoots of four different genotypes. However, the *NHX* family transcripts were markedly up-regulated in all genotypes, but there are significant differences between different genotypes. *OsSOS1* was significantly up-regulated in roots, especially in *JYGY-1*genotype.

**Conclusions:**

The results showed that different genotypes had different germination and nutrient survival under salt stress, which was related to the difference of ion content and the difference of a series of ion transport gene expression. At the same time this study will provide new insight into the similarities and differences in ion homeostasis and gene regulatory mechanisms between weedy and cultivated rice under salt stress, which can aid in novel rice breeding and growth strategies.

**Electronic supplementary material:**

The online version of this article (10.1186/s12870-018-1586-9) contains supplementary material, which is available to authorized users.

## Background

Salt stress is one of the most serious abiotic stresses that affects plant natural productivity and causes significant crop loss worldwide. Sodium (Na^+^) and potassium (K^+^) ions were present in the ocean during early life evolution, but only K^+^ ions maintain electrolyte and osmotic balance in the cells of organisms [1]. One of the many important physiological changes during early plant cell evolution was the ability to adapt to low levels of Na^+^ and K^+^ intermediates. This evolution has led to the ability for plants to assimilate nutrients from low ionic concentrations in their growth medium, but they often cannot tolerate high Na^+^ concentrations [1]. The effects of salinity are diverse, but Na^+^ toxicity is one of the primary mechanisms of cell damage in most salt-sensitive plants, whereas K^+^ is an essential ion [2]. The cytosol of plant cells normally contains 100–200 mM K^+^ and 1–10 mM Na^+^; this Na^+^/K^+^ ratio is optimal for many metabolic cell functions [2]. Therefore, it is essential that the cytosol maintain a low concentration of Na^+^ or a low Na^+^/K^+^ ratio in cells when under salt stress.

**Fig. 1:**
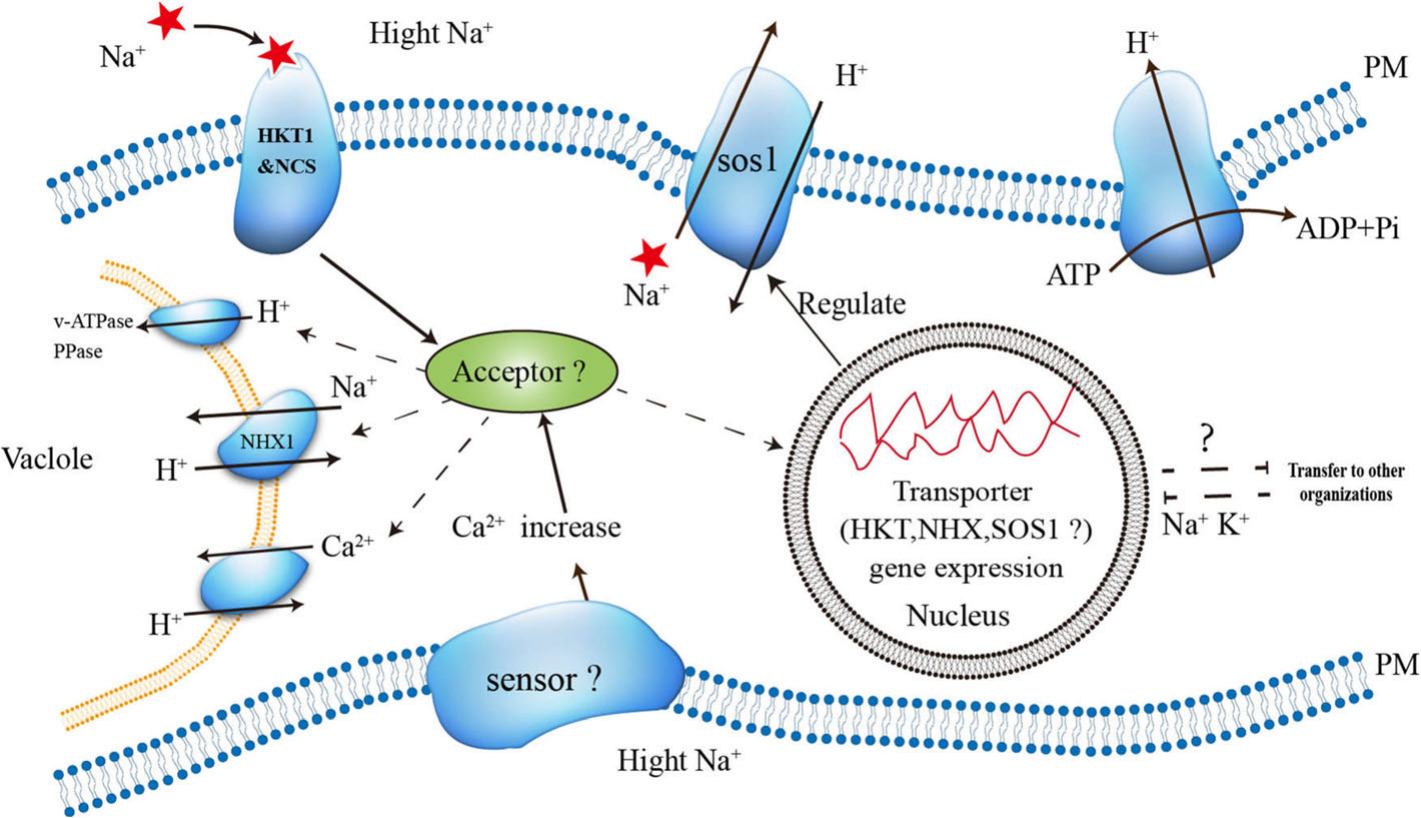
Transport regulatory mechanisms of Na^+^ in plants under salt stress. High-affinity K^+^ transporter (*HKT1*) mediate Na^+^-specific transport or Na^+^-K^+^ transport, and play a key role in regulation of Na^+^ homeostasis. Salt overly sensitive 1 (*SOS1*) plasma membrane Na^+^/H^+^ antiporter exports intracellular Na^+^ to extracellular. Sodium-hydrogen exchanger (*NHX1*)in the tonoplast can sequestrate Na^+^ into the vacuole. While balancing the Na^+^ in the cytoplasm, *SOS1* and *NHX1* convert H^+^ in the extracellular and vacuolar into the cytoplasm, and excess H^+^ in the cytoplasm are transported to extracellular by consuming energy (ATP is converted to ADP + Pi)

For many plants, leaves that develop photosynthesis and other metabolic processes are the main sites of Na^+^ toxicity [3]. Some mechanisms to alleviate Na^+^ toxicity in leaves have been discovered and/or proposed in rice. They include limiting the absorption of Na^+^ from the soil, reducing the transport of Na^+^ to the xylem, storing Na^+^ in the lower part of the leaf (such as the sheath), isolating Na^+^ into the vacuole, and cycling Na^+^ from shoots to roots [4]. Intracellular Na^+^ is transported out of the cell via *SOS1* transporter [5], or into the root xylem by the high-affinity potassium transporter (*HKT1;4, HKT1;5*), which is essential for relieving Na^+^ toxicity in stems. The Na^+^ can also be sequestrated into the vacuole via the sodium-hydrogen exchanger (*NHX1*) in the tonoplast [6] (Fig. 1).

The rice cultivar *Nipponbare* (*Oryza sativa* L.) genome contains seven *HKT* genes and two *HKT* pseudogenes [1]. The salt tolerance of plants may depend on the *HKT* transporter, which play a key role in regulating Na^+^ homeostasis because it mediate Na^+^-specific or Na^+^-K^+^ transport [1]. Members of the *HKT* transporter family are highly conserved in both monocots and dicots such as *AtHKT1;4* in *Arabidopsis* and *OsHKT1;5* in *Oryza sativa*, which have similar functions in their respective plant species. These transporters reduce Na^+^ transport to shoots and actively regulate salt tolerance [7]. The *TmHKT1;4* and *TmHKT1;5* proteins have been reported in wheat with similar mechanisms [8]. The sequestering of Na^+^ lowers the cytoplasmic Na^+^ concentration and also helps to maintain the osmotic adjustment of water absorption in the salt solution, which indicates that the separation of Na^+^ into vacuoles is the basic strategy for plant salt tolerance [9]. The *OsNHX1, OsNHX2, OsNHX3, OsNHX4,* and *OsNHX5* antiporter genes have been identified in rice [10]. The relative abundance of *NHX* transcripts was determined by RNA gel blotting and RT-PCR and the results showed that *NHX1* and *NHX2* were abundant and widely distributed in plant tissues, and the same effectors (NaCl, KCl, LiCl, osmotic pressure and abscisic acid) have a certain accumulation [11] (Fig. 1). Although the mechanisms of ion-homeostasis and transport regulation under salt stress have been studied extensively in cultivated rice, they have not been studied in weedy rice (*O. sativa* L.).

Weedy rice is the result of de-domestication of rice, so the genetic background, morphology and growth behavior are similar to cultivated rice [12]. However, there will be differences in stress tolerance during de-domestication, such as weedy rice has cold tolerance at the seedling stage, rice blast resistance, high salinity and drought tolerance, as well as positive germination characteristics [13]. Weedy rice has become one of the most stubborn and harmful weeds in rice-growing regions worldwide may be due to its stronger competitiveness and stress tolerance. Recent investigation found that weedy rice has infested rice field all over china over the past 5 years, and the area has grown to more than three million hectares [14]. Not only that, weedy rice has a serious negative impact on rice production areas in other countries. Approximately 50% of US rice is produced in Arkansas where 60% of the rice fields are infested at different levels by weedy rice [15]. A similar proportion of rice fields is infested by weedy rice in Costa Rica [16]. Therefore, weedy rice is considered an important germplasm resource to improve rice varieties, and the screening of salt-tolerant weedy rice based on Na^+^/K^+^ transport regulatory systems, and further identifying and transferring useful genes from weedy rice to cultivated rice varieties, might play vital roles in the improvement of cultivated rice’s tolerance to salt stress.

We observed that the weedy rice accession *JYGY-1* is salt-tolerant, whereas the accession *JYFN-4* is relatively salt-sensitive. Furthermore, the two rice cultivars *Nipponbare* and *9311* were compared to the weedy rice accessions with the following objectives: to (i) investigate the differences in the phenotypes under salt stress among the weedy and cultivated rice lines; (ii) study the ion homeostasis among weedy and cultivated rice lines under salt stress; (iii) identify the homology levels of the Na^+^/K^+^ transport genes between weedy and cultivated rice lines; and (iv) determine the Na^+^/K^+^ transport regulation-related gene expression levels and its contribution to salt damage resistance/protection when weedy and cultivated rice under salt stress.

## Results

### Effects of salt stress on seed germination and seedling growth of weedy rice and cultivated rice

**Fig. 2:**
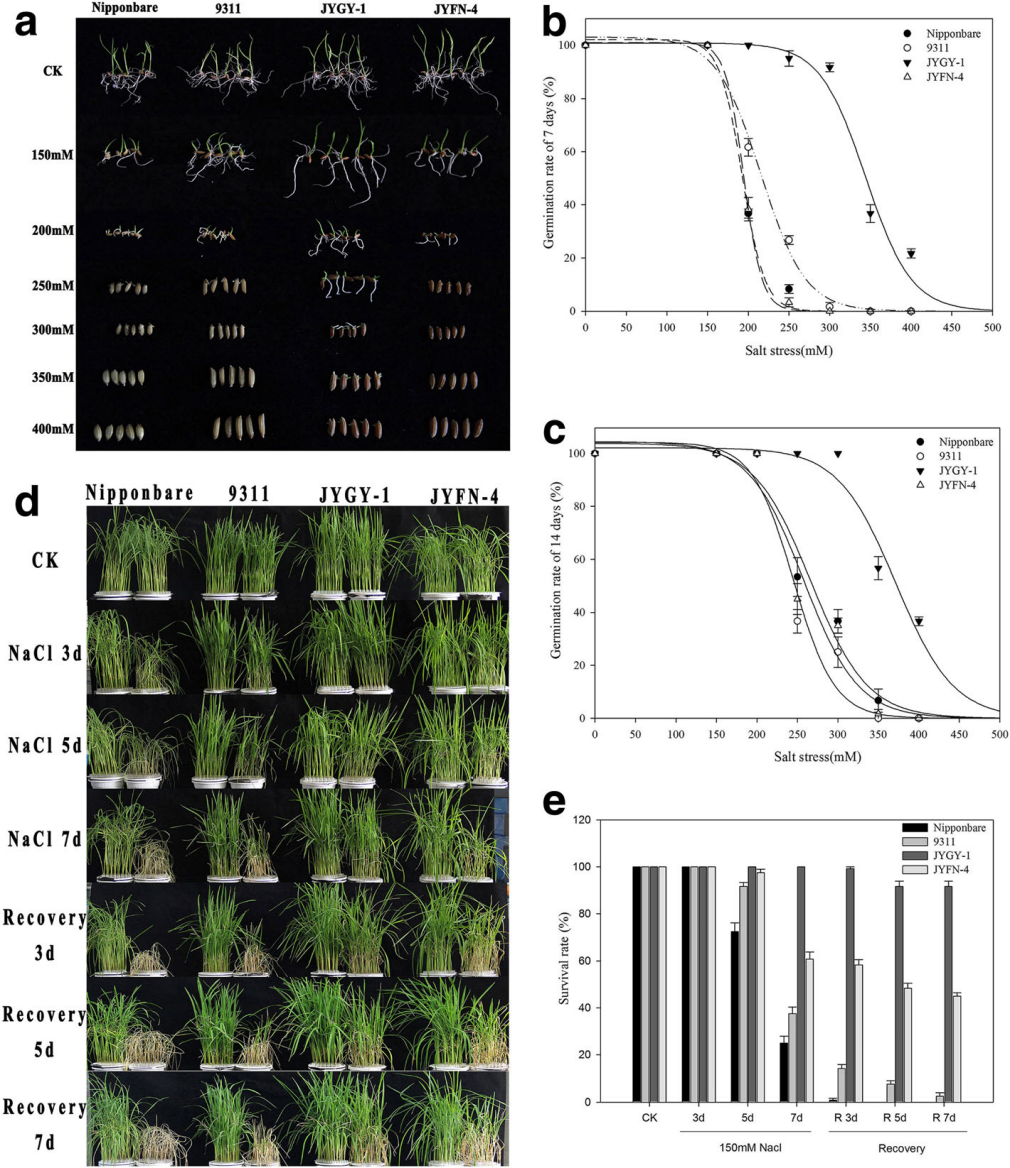
Seed germination and seedling growth of different weedy rice and cultivated rice genotypes under salt stress. Seed germination after 14 d in different salt stress conditions (**a**). **b** and (**c**) are graphs representing the seed germination rates for the different genotypes under salt stress after 7 and 14 d, respectively. The seedlings were treated for 7 d with 150 mM NaCl and allowed to recover for 7 d (**d**). Graphical representation of the survival rate of seedlings treated for 7 d with 150 mM NaCl and allowed to recover for 7 d (**e**)

Among the four different genotypes of weedy and cultivated rice, the seed germination rate decreased to different degrees as the salt concentration increased. At 200–250 mM NaCl, the seed germination in the *JYGY-1* population was significantly higher than that in the other three genotypes (Fig. 2a). The seed germination in *Nipponbare*, *9311*, and JYGN-4 lines was inhibited by a 7–14 d treatment of 400 mM NaCl, while *JYGY-1* seed was still able to germinate at this salt concentration (Fig. 2b, c). To further analyze the response of rice seedlings to salt stress, we used a hydroponic culture system to grow rice seedlings in 150 mM NaCl, which has been shown to be the best condition for observing salt stress in liquid culture media growth conditions, as well as being highly reproducible. At this level of salt stress, morphological damage was evident after exposure of 2 week old seedlings to salt stress for 3 days. The most obvious morphological change appeared on the seventh day after salt stress treatment; the height of *Nipponbare*, *9311*, and *JYFN-4* plants was significantly reduced, and the leaves gradually yellowed. In contrast, the *JYGY-1* population was almost unaffected (Fig. 2d). After 3–5 d of recovery, the *Nipponbare* and *9311* rice genotypes gradually died, while the older leaves of the weedy rice genotypes *JYGY-1* and *JYFN-4* began to yellow to different degrees. However, after 7 d of recovery, weedy rice populations were still able to grow new leaves (Fig. 2d, e). Thus, the survival rates were rated as *JYGY-1* > *JYFN-4* > *9311* > *Nipponbare*, indicating that the weedy rice genotype *JYGY-1* had the highest salt tolerance during both seed germination and seedling growth.

### Ion content changes in roots and shoots of weedy and cultivated rice under salt stress

**Fig. 3:**
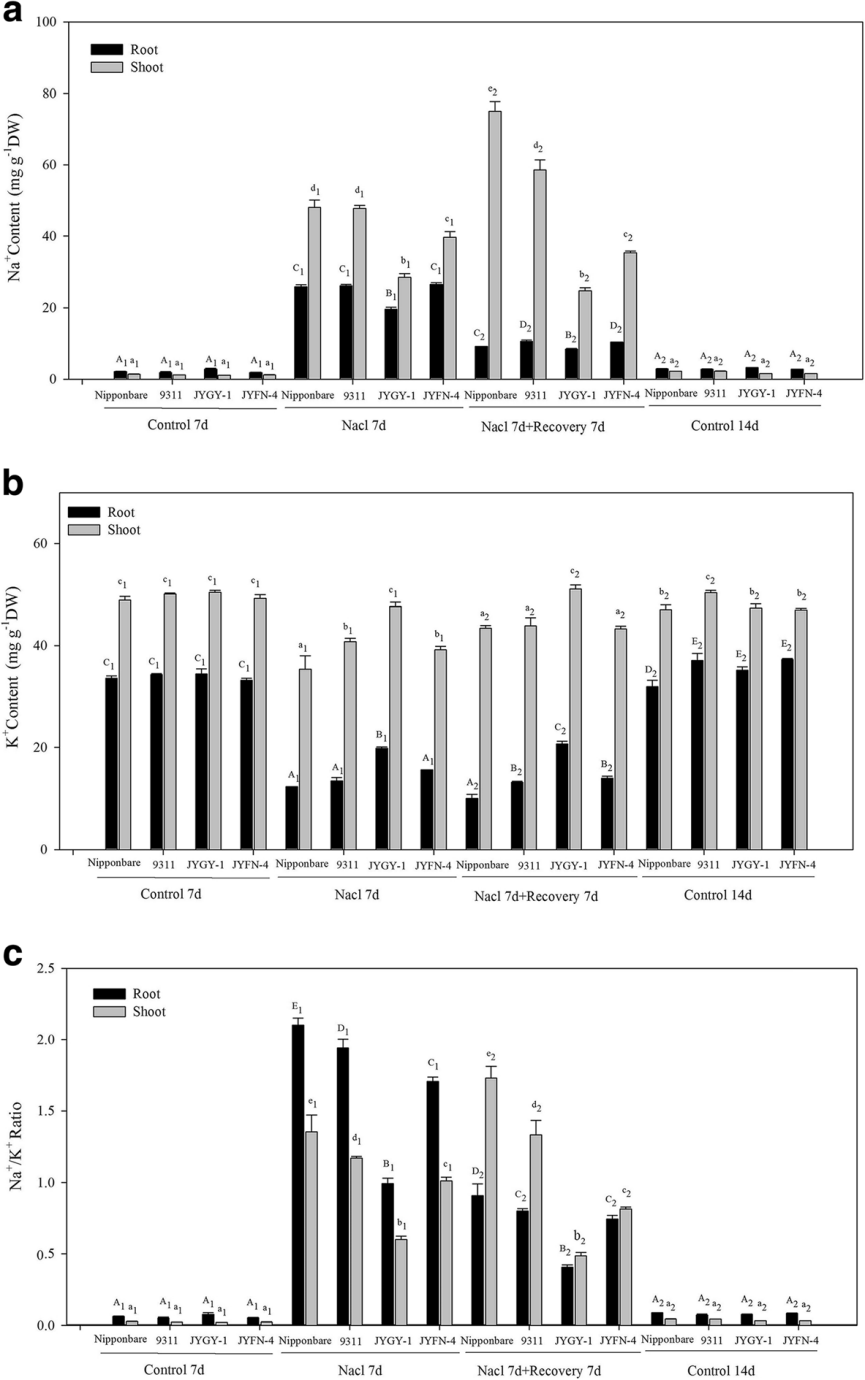
The Na^+^ and K^+^ content in roots and shoots of different rice genotypes were determined after 7 d of 125 mM salt stress and recovery. **a** Na^+^ content in roots and shoots. **b** K^+^ content in roots and shoots. **c** Na^+^/ K^+^ ratio in roots and shoots. Different letters indicate statistically significant differences between treatments the different treatments (*P* < 0.05 by Duncan’s Multiple Range test)

Under salt stress, the content of Na^+^ and K^+^ in the shoots and roots was significantly changed in all genotypes. After 7 days of salt stress treatment, the Na^+^ content increased significantly compared to the control, while K^+^ content decreased. Under salt stress, the Na^+^ content in roots of the four genotypes, *Nipponbare*, *9311*, *JYGY-1*, and *JYFN-4* increased approximately by 12.1, 14.6, 7.3 and 14.8-fold, respectively, and in shoots the Na^+^ content increased by 34.3, 42.0, 26.3, 33.8-fold, respectively. The K^+^ content of the roots decreased by 2.7, 2.5, 1.7, and 2.1-fold, and decreased by 1.4, 1.2, 1.1 and 1.3-fold in the shoots of *Nipponbare*, *9311*, *JYGY-1*, and *JYFN-4*, respectively (Fig. 3a, b). The Na^+^ content in *JYGY-1* was significantly lower than that in the other three genotypes, but the K^+^ content was significantly higher. After 7 days of nutrient recovery, the Na^+^ in the roots of all four genotypes decreased significantly and only the shoots of *JYGY-1* and *JYFN-4* genotypes showed decreased Na^+^ levels (Fig. 3a). The K^+^ concentrations in roots and shoots remained almost unchanged in all genotypes after recovery (Fig. 3b). The ratio of Na^+^/K^+^ in roots and shoots was significantly increased after salt stress treatment in the order of *Nipponbare* > *9311* > *JYFN-4* > *JYGY-1*. After recovery, in addition to the rise of the Na^+^/K^+^ ratio in the shoots of the rice populations, the Na^+^/K^+^ ratio in weedy rice and in the roots of the rice populations were significantly decreased (Fig. 3c). The Na^+^ and K^+^ measurements were consistent with the phenotypes of the different genotypes under salt stress. Under salt stress, the uptake and transportation of other ions in addition to Na^+^ and K^+^ were also sensitive to salt stress treatment. In this study, Mn^2+^, Cu^2+^, Zn^2+^, Ca^2+^, Mg^2+^, P, and Fe^2+^ content in the roots and shoots of the four different genotypes changed to differing degrees after salt stress treatment and recovery. In the roots and shoots of all four genotypes, Mn^2+^, Mg^2+^, and Fe^2+^ content decreased after salt stress treatment and increased after recovery. The content of Ca^2+^ in both roots and shoots increased after salt stress treatment and recovery. In all genotypes, the P and Cu^2+^ content in the shoots decreased after salt stress treatment, while in roots they increased after salt stress and decreased after recovery. Additionally, the Zn^2+^ content in the shoots increased after salt stress and recovery, while in the roots did not change or decreased at first and then increased (Table 1). Therefore, under salt stress, salt-tolerant weedy rice population *JYGY-1* had an improved adaptability to salt stress conditions mainly through the regulation of its Na^+^ and K^+^ balance. The regulation of the absorption of Ca^2+^, Mn^2+^, Mg^2+^, P, Cu^2+^, Zn^2+^, and Fe^2+^ iron may also play an important role in increased salt tolerance or the repair from salt poisoning in the plant.

**Table 1:** Ion concentrations of root and shoot of cultivated and weedy rice under salt stress

Ion	Salt stress (125 mM)	Ion content							
		*Nipponbare* ^*^		*9311*		*JYGY-1*		*JYFN-4*	
		Shoot	Root	Shoot	Root	Shoot	Root	Shoot	Root
Ca^2+^	Control 7 d^**^	4.09 ± 0.17^b***^	1.41 ± 0.04^c^	4.23 ± 0.16^b^	1.48 ± 0.1^c^	3.67 ± 0.07^c^	0.78 ± 0.11^c^	4.24 ± 0.21^b^	0.92 ± 0.03^c^
	NaCl 7 d	4.5 ± 0.16^b^	1.64 ± 0.02^bc^	4.38 ± 0.12^b^	1.75 ± 0.13^c^	4.34 ± 0.07^b^	1.36 ± 0.04^b^	4.57 ± 0.22^b^	1.08 ± 0.06^c^
	Recovery 7 d	5.36 ± 0.22^a^	3.3 ± 0.22^a^	5.29 ± 0.26^a^	3.66 ± 0.21^a^	5.32 ± 0.05^a^	1.99 ± 0.08^a^	5.09 ± 0.04^a^	2.22 ± 0.07^a^
	Control 14 d	3.49 ± 0.02^c^	1.83 ± 0.09^b^	3.64 ± 0.07^c^	2.22 ± 0.05^b^	3.41 ± 0.05^c^	1.55 ± 0.06^b^	3.67 ± 0.03^c^	1.97 ± 0.06^b^
Mg^2+^	Control 7 d	7 ± 0.1^b^	1.36 ± 0.01^c^	8.65 ± 0.44^bc^	1.74 ± 0.07^b^	6.02 ± 0.13^c^	1.49 ± 0.05^b^	6.16 ± 0.14^b^	0.93 ± 0.04^c^
	NaCl 7 d	5.19 ± 0.07^c^	1.26 ± 0.02^c^	7.61 ± 0.18^c^	1.33 ± 0.02^c^	4.72 ± 0.12^d^	0.5 ± 0.03^c^	5.52 ± 0.24^b^	0.44 ± 0.02^d^
	Recovery 7 d	8.43 ± 0.15^a^	2.06 ± 0.05^a^	9.29 ± 0.25^ab^	1.77 ± 0.01^b^	7.01 ± 0.06^b^	1.54 ± 0.03^b^	8.05 ± 0.09^a^	1.63 ± 0.01^b^
	Control 14 d	8.77 ± 0.25^a^	1.8 ± 0.1^b^	10.04 ± 0.35^a^	2.14 ± 0.05^a^	8.03 ± 0.09^a^	2.3 ± 0.01^a^	8.3 ± 0.17^a^	2.2 ± 0.04^a^
P	Control 7 d	15.34 ± 0.73^ab^	10.11 ± 0.4^b^	16.06 ± 0.46^a^	9.55 ± 0.26^b^	14.06 ± 0.68^a^	5.07 ± 0.2^c^	15.17 ± 0.36^a^	8.56 ± 0.28^bc^
	NaCl 7 d	13.52 ± 0.67^b^	12.33 ± 0.25^a^	13.41 ± 0.33^b^	13.33 ± 0.79^a^	12.29 ± 0.56^b^	9.44 ± 0.17^a^	14.19 ± 0.09^bc^	10.4 ± 0.4^a^
	Recovery 7 d	14.07 ± 0.54^b^	10.13 ± 0.33^b^	11.22 ± 0.64^c^	13.07 ± 0.43^a^	13.26 ± 0.11^ab^	8.96 ± 0.35^a^	14.9 ± 0.35^ab^	9.5 ± 0.23^ab^
	Control 14 d	16.36 ± 0.63^a^	8.63 ± 0.27^c^	14.95 ± 0.34^a^	8.3 ± 0.15^b^	14.49 ± 0.15^a^	7.51 ± 0.12^b^	13.33 ± 0.13^c^	7.98 ± 0.25^c^
Fe^2+^	Control 7 d	0.27 ± 0.01^bc^	2.24 ± 0.05^a^	0.26 ± 0.01^b^	2.62 ± 0.02^a^	0.27 ± 0^a^	3.01 ± 0.02^a^	0.3 ± 0.02b^c^	2.49 ± 0.16^a^
	NaCl 7 d	0.25 ± 0.01^c^	1.98 ± 0.08^b^	0.24 ± 0.01^c^	2.11 ± 0.02^c^	0.24 ± 0.01^b^	1.24 ± 0.04^c^	0.28 ± 0.01^c^	1.88 ± 0.03^b^
	Recovery 7 d	0.3 ± 0.02^a^	2.18 ± 0.04^a^	0.28 ± 0.02^a^	2.58 ± 0.06^a^	0.28 ± 0.01^a^	1.53 ± 0.06^b^	0.32 ± 0.01^ab^	2.12 ± 0.06^b^
	Control 14 d	0.28 ± 0.01^ab^	1.49 ± 0.01^c^	0.24 ± 0.01^c^	2.41 ± 0.02^b^	0.21 ± 0.01^c^	1.6 ± 0.03^b^	0.33 ± 0.01^a^	1.3 ± 0.01^c^
Mn^2+^	Control 7 d	1389 ± 50.54^a^	142.67 ± 5.81^b^	938.33 ± 18.7^a^	137.33 ± 1.33^b^	816.33 ± 4.1^a^	68 ± 0^a^	949.67 ± 68.63^a^	61.33 ± 4.81^a^
	NaCl 7 d	640 ± 28.57^b^	54.67 ± 1.33^c^	750 ± 12.77^b^	66.67 ± 3.53^c^	560.67 ± 19.36^b^	13.33 ± 1.33^d^	567.33 ± 13.48^b^	29.33 ± 1.33^b^
	Recovery 7 d	739.33 ± 15.1^b^	378.67 ± 7.42^a^	760 ± 17.21^b^	394.67 ± 12.7^a^	825.67 ± 17.32^a^	46.67 ± 2.67^b^	911.67 ± 12.47^a^	57.33 ± 1.33^a^
	Control 14 d	740.33 ± 7.8^b^	60 ± 0^c^	746.33 ± 10.8^b^	76 ± 4^c^	606.33 ± 7.13^b^	38.67 ± 1.33^c^	524.67 ± 9.24^b^	34.67 ± 3.33^b^
Cu^2+^	Control 7 d	20.33 ± 1.2^a^	193.33 ± 7.1^ab^	23.67 ± 1.67^a^	253.33 ± 1.3^c^	22.33 ± 0.88^a^	193.33 ± 1.3^b^	19 ± 1.53^a^	172 ± 2.31^c^
	NaCl 7 d	19.67 ± 1.2^a^	210.67 ± 9.33^a^	21.33 ± 0.67^a^	349.33 ± 10.4^a^	13.33 ± 0.33^b^	261.3 ± 6.67^a^	15.33 ± 0.88^b^	208 ± 4^b^
	Recovery 7 d	16 ± 0.58^b^	177.33 ± 9.61^b^	21 ± 1^a^	280 ± 8.33^b^	14.33 ± 0.67^b^	214.7 ± 10.4^b^	16.33 ± 0.33^ab^	230.67 ± 7.42^a^
	Control 14 d	14 ± 0.58^b^	132 ± 0^c^	20.33 ± 0.33^a^	244 ± 4.62^c^	14.67 ± 0.33^b^	133.33 ± 4.8^c^	16 ± 0.58^ab^	145.33 ± 5.33^d^
Zn^2+^	Control 7 d	74.67 ± 2.96^a^	70.67 ± 8.11^b^	40.67 ± 1.33^c^	69.33 ± 7.42^b^	38.33 ± 0.33^c^	76 ± 2.31^b^	56 ± 2^c^	141.33 ± 5.81^b^
	NaCl 7 d	77.33 ± 0.88^a^	64 ± 2.31^b^	54 ± 1.53^b^	52 ± 4.62^b^	42.33 ± 0.88^b^	60 ± 8.33^b^	66 ± 2.08^b^	104 ± 6.11^c^
	Recovery 7 d	82.33 ± 6.12^a^	112 ± 4^a^	61 ± 1.73^a^	128 ± 10.07^a^	60 ± 1.53^a^	97.33 ± 8.74^a^	75.67 ± 2.73^a^	176 ± 6.11^a^
	Control 14 d	57.67 ± 3.28^b^	82.67 ± 9.61^b^	42 ± 1.53^c^	66.67 ± 4.81^b^	38.33 ± 1.45^c^	60 ± 2.31^b^	37.67 ± 0.88^d^	178.67 ± 3.53^a^

The samples that seedlings had three fully expanded leaves, were harvested after treated with nutrient solution containing 125 mM NaCl for 1 weeks, and recovered with IRRI nutrient solution for 1 weeks, respectively. The ion content of Ca^2+^, Mg^2+^, P and Fe^2+^ were mg/g, and Mn^2+^, Cu^2+^ and Zn^2+^ were mg/kg, indicated by dry weight

*Nipponbare is a japonica rice cultivar, *9311* an indica rice cultivar, *JYGY-1* a salt-tolerant weedy rice genotype, and *JYFN-4* a salt-sensitive weedy rice genotype

**Control 7 d and Control 14 d indicate control conditions (no stress). NaCl 7 d indicates salt stress treatment for 7 days. Recovery 7 d indicates that the first salt stress treatment lasted 7 days and then recovery in normal nutrient conditions for 7 days

*** “±” indicate standard error and different letters (a-d) indicate statistically significant differences between treatments the different treatments (*P* < 0.05 by Duncan’s Multiple Range test)

### Comparison of homology in ion transport-related genes between weedy rice and rice

The *HKT* and *NHX* gene families, and the *SOS1* gene play an important role in the transport of ions such as sodium, potassium, and calcium [1, 5, 24]. When comparing the gene and amino acid sequences, the homology level was greater than 98% between the cultivated and weedy rice genotypes (Additional file 1: Table S1). Among them, the gene with the most amino acid change between weedy rice and rice is *NHX4*, as there are eight amino acid changes identified, followed by *OsHKT2;2*, *OsHKT2;3*, *OsHKT2;4*, *OsHKT1;5*, Os*NHX2*, *OsHKT1;1*, Os*SOS1* and *OsHKT1;4* (Additional file 2: Figure S1). There are seven *HKT* genes and two *HKT* pseudogenes (*OsHKT2;2* and *OsHKT1;2*) in *Nipponbare* rice, but some salt-tolerant rice varieties such as indica rice that have a complete *OsHKT2;2* gene [25]. It is noteworthy that these two *HKT* genes exist in weedy rice and have high homology with indica rice (Additional file 2: Figure S1). Gene homology study results confirms that weedy rice is an important germplasm resource for rice cultivation improvement.

### Comparison of the gene expression of ion transport-related genes between weedy rice and cultivated rice genotypes under salt stress

**Fig. 4:**
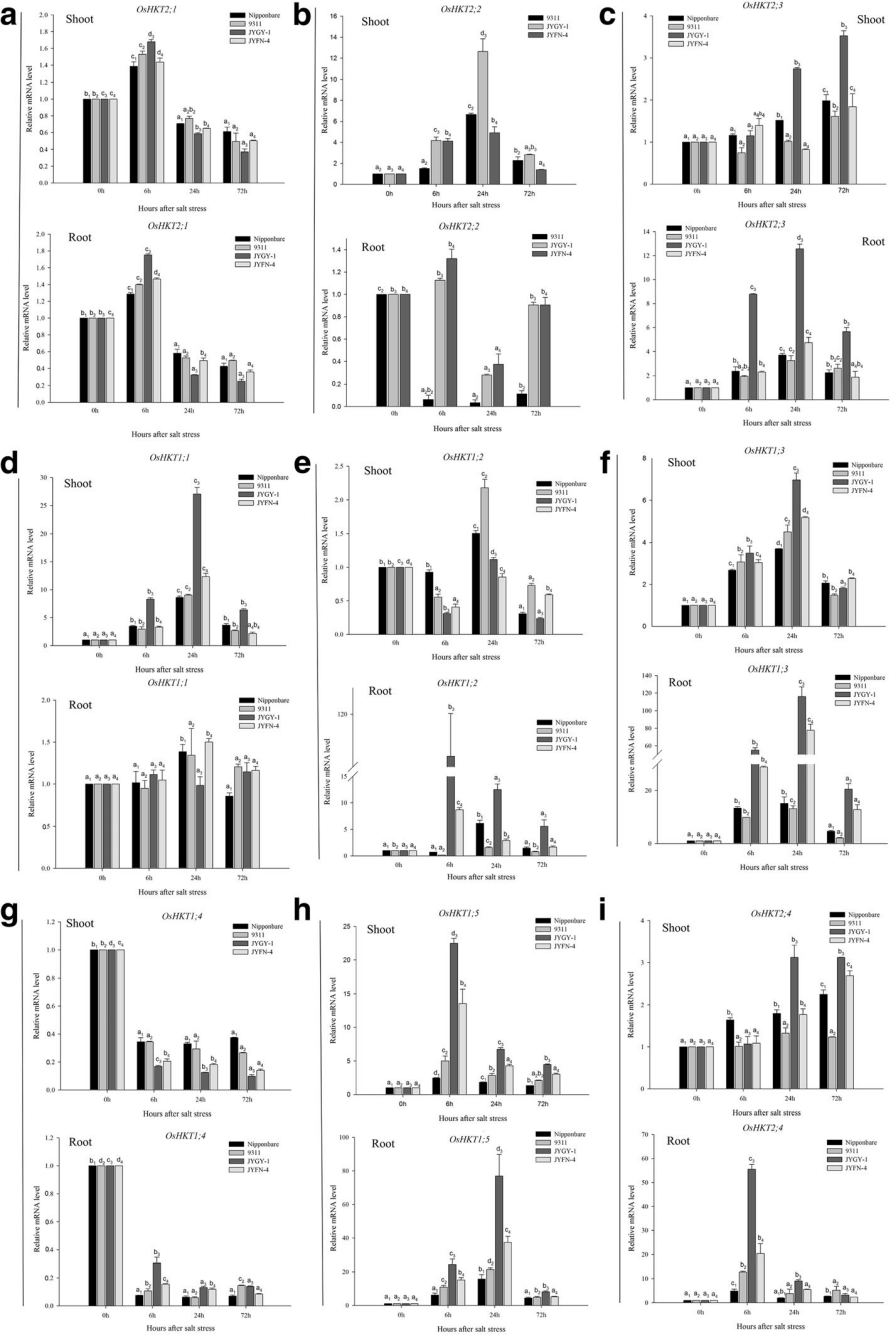
Expression analysis of the *HKT* family by quantitative real-time PCR amplification of RNA from root and shoot tissue from weedy rice (*JYGY-1* and *JYFN-4*) and rice (*Nipponbare* and *9311*) genotypes. (a)* OsHKT2;1*, (b) *OsHKT2;2*, (c) *OsHKT2;3*, (d) *OsHKT1;1*, (e) *OsHKT1;2*, (f) *OsHKT1;3*, (g) *OsHKT1;4*, (h) *OsHKT1;5*, (i) *OsHKT2;4*. The number of hours (6, 24, and 72 h) elapsed after growing plants under salt stress (125 mM NaCl) conditions is indicated. Amplification was performed with specific primers for HKT family and compared to their expression under control conditions (no stress). Different letters indicate statistically significant differences between treatments the different treatments (*P* < 0.05 by Duncan’s Multiple Range test)

Based on the qRT-PCR results, we wanted to confirm that the changes in gene expression levels were related to ion transport regulation under salt stress. The expression of all nine genes was visualized using real-time PCR (Fig. 4). Among the nine *HKT* genes identified, *OsHKT2;1* was down-regulated after 24 h and *OsHKT1;4* at all the times under salt stress. After 24 h of salt stress treatment, the expression of *OsHKT2;1* began to decrease, and the *JYGY-1* population showed the most significant decline in *OsHKT2;1* expression, with more than a 2-fold and 4-fold reduction in shoot and root tissues, respectively (Fig. 4a). In all genotypes tested, the expression of *OsHKT2;2* was up-regulated in the shoots, and *JYGY-1* had more than a 12-fold increase in *OsHKT2;2* expression; however, in the roots, this gene was down-regulated, and in the *9311* population, this gene was down-regulated more than 30-fold (Fig. 4b). The *OsHKT2;3, OsHKT1;1, OsHKT1;3, OsHKT1;5*, and *OsHKT2;4* genes were all significantly up-regulated in all genotypes under salt stress after 6 h except the *OsHKT2;3* of 9311 in the shoots was down regulated and *OsHKT1;1* was little upregulated in the roots of any of the tested genotypes. The *JYGY-1* genotype had the most significant level of up-regulation, *JYFN-4* genotype followed by and the *Nipponbare* genotype had the lowest level of *OsHKT1;1* gene expression (Fig. 4). The expression of *OsHKT1;2* was down-regulated under salt stress after 6 h in the shoots, but in the roots, it was up-regulated, particularly in the *JYGY-1* population, which showed more than a 110-fold change in gene expression (Fig. 4e). The *OsHKT1;4* expression was down-regulated more than 10-fold after 6 h, with the *JYGY-1* population having the largest decrease in gene expression in the shoots, but not in root tissue (Fig. 4g).

**Fig. 5:**
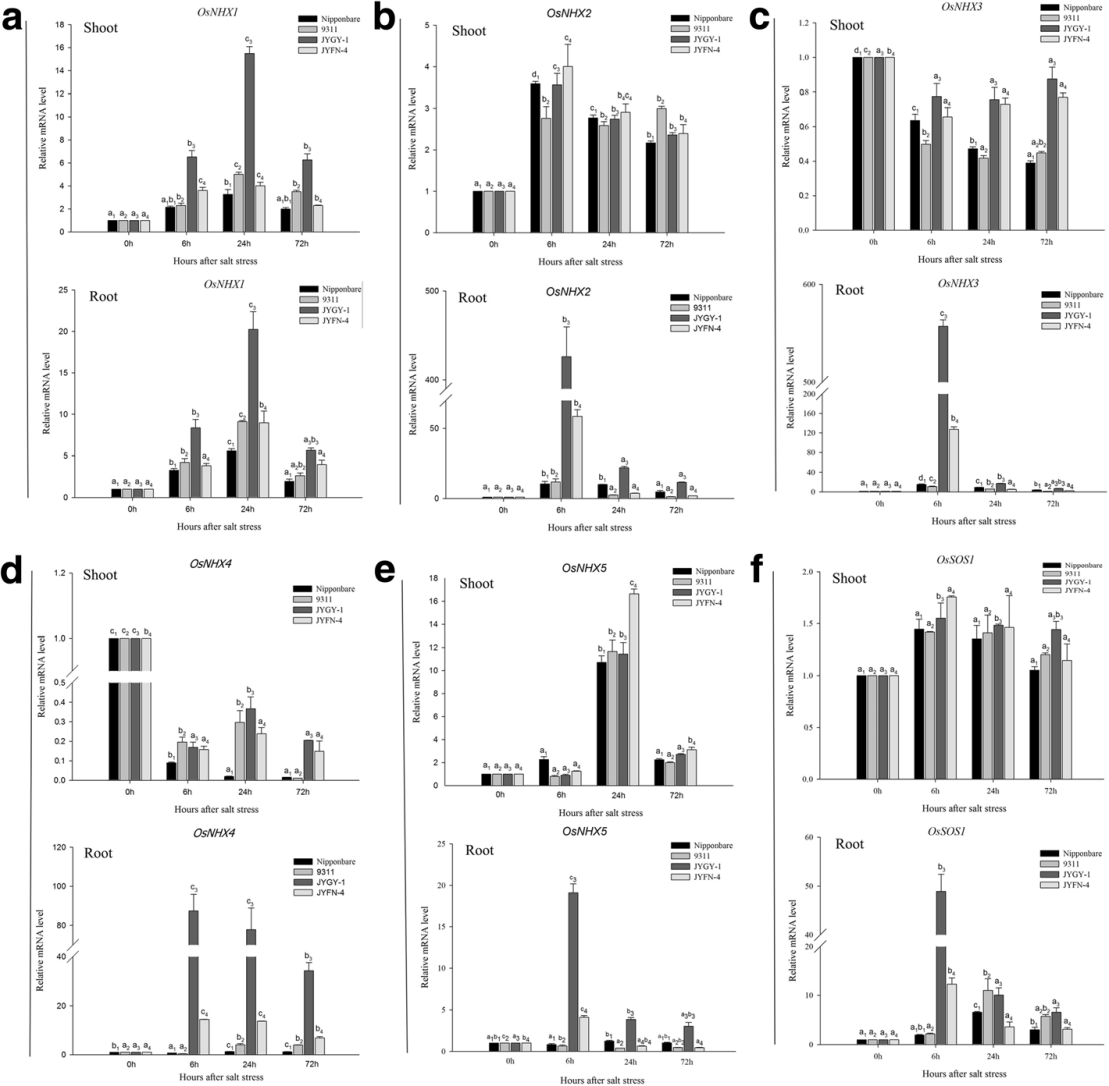
Expression analysis of the *NHX* family and *OsSOS1* genes by quantitative real-time PCR amplification of RNA from root and shoot tissue from weedy rice (*JYGY-1* and *JYFN-4*) and rice (*Nipponbare* and *9311*) genotypes. (a) *OsNHX1*, (b) *OsNHX2*, (c) *OsNHX3*, (d) *OsNHX4*, (e) *OsNHX5*, (f) *OsSOS1*. The number of hours (6, 24, and 72 h) elapsed after growing plants under salt stress (125 mM NaCl) conditions is indicated. Amplification was performed with specific primers for *NHX* family and *OsSOS1* genes and compared to their expression under control conditions (no stress) Different letters indicate statistically significant differences between treatments the different treatments (*P* < 0.05 by Duncan’s Multiple Range test)

Several of the *OsNHX* gene family members were significantly up-regulated under salt stress, while *OsNHX3* and *OsNHX4* were down-regulated in roots (Fig. 5). *OsNHX1* reached a maximum level of expression 24 h after salt stress, and the *JYGY-1* population had the highest level of up-regulation in both the shoots and roots, which increased > 14 and > 20-fold, respectively (Fig. 5a). The expression of *OsNHX2* and *OsNHX3* genes was up-regulated and down-regulated approximately 3-fold and 2-fold after salt treatment 6 h in the shoots, respectively; however, in the roots the expression of these genes increased > 420-fold and 550-fold in the *JYGY-1* population, while in the other populations they increased < 60-fold and 130-fold, respectively (Fig. 5b, c). The expression of *OsNHX4* in the shoots was down-regulated, and in the roots, it was up-regulated after 6 h for the genotypes tested in this study. In *JYGY-1*, *OsNHX4* expression decreased < 5-fold and increased > 90-fold in shoots and roots, respectively (Fig. 5d). The expression of *OsNHX5* in shoot tissue increased > 10-fold after salt treatment 24 h in the four genotypes; however, in the roots it increased > 19-fold in *JYGY-1* and < 5-fold after 6 h in other genotypes (Fig. 5e). The expression of *OsSOS1* did not change significantly in the shoots after salt stress treatment, but it was significantly up-regulated in the roots of all genotypes. The maximum *OsSOS1* expression in weedy rice populations (*JYGY-1* and *JYFN-4*) was achieved 6 h after salt stress, and was approximately 49-fold and 12-fold, respectively. However, the cultivated rice populations (*Nipponbare* and *9311*) achieved maximum levels of gene expression in 24 h that were approximately 6-fold and 11-fold, respectively (Fig. 5f).

## Discussion

Rice is the main food crop grown worldwide and salt stress is one of the major abiotic stress factors restricting its yield. Weedy rice has become a malignant weed in cultivated rice fields and has a very high homology to cultivated rice lines. This homology may make weedy rice an important germplasm resource for the improvement of current rice varieties if its genes for cold tolerance at the seedling stage, blast resistance, and high salinity and drought tolerance can be characterized [13]. Our studies have found that weedy rice has a strong salt tolerance, so it is important to study the differences in ion homeostasis and transport regulation mechanisms between weedy rice and traditionally cultivated rice lines under salt stress.

### Weedy rice has stronger salt tolerance than cultivated rice during seed germination and seedling growth

Weedy rice has acquired many characteristics of weed in the process of de-domestication, which makes it have stronger environmental adaptability [12]. A stronger stress tolerance might be one of the reasons for the occurrence of weedy rice in cultivated rice fields. Weedy rice has also been reported to have a strong tolerance to drought and low temperature stress [13]. Our study further confirmed that weedy rice has stronger salt tolerance than cultivated rice by salt stress determination of 74 weedy rice populations collected. This is consistent with a previous study that showed that weedy rice was tolerant to a 0.5% saline-alkaline solution [26]. Although the genotype *JYGY-1* and *JYFN-4* all showed stronger salt tolerance than the cultivated rice genotypes *Nipponbare* and *9311* in the reproductive growth stage of seedlings, the genotype *JYFN-4* performed basically consistent with cultivated rice in the seed germination stage (Fig. 2a, b). The difference in genotype *JYFN-4* tolerance during seed germination and vegetative growth under salt stress indicates that salt tolerance in these two stages can be inconsistent. Previous studies have found that during rice de-domestication not only endows cold tolerance, blast resistance, high salinity and drought tolerance on weedy rice at the seedling stage, but also gives its seeds positive germination characteristics [13]. Therefore, we can speculate that in the process of rice de-domestication to weedy rice, the salt tolerance of the seed stage and vegetative growth stage may not be obtained at the same time, for example, genotype *JYFN-4*. Thus, weedy rice is a good material in which to study the mechanism of salt tolerance and improve on the salt tolerance in cultivated rice lines.

### Weedy rice enhances salt tolerance by regulating Na^+^/K^+^ ratios and other ion absorption under salt stress

Numerous studies have shown that salt tolerance is ultimately manifested in plants through several physiological processes, including Na^+^ uptake and exclusion, ion homeostasis, especially between Na^+^ and K^+^, and partitioning [7]. Various studies have shown that plants increase Na^+^ uptake and reduce K^+^ uptake under salt stress [9, 25]. The K^+^ ions are beneficial to plants, and by increasing K^+^ content, plants can reduce the absorption of Na^+^ ions to a certain extent, thus reducing the Na^+^/K^+^ ratio. This is consistent with our findings that the salt-tolerant genotype *JYGY-1* has the lowest Na^+^/K^+^ ratio (Fig. 3). In addition, the negative relationship between Mg^2+^ and K^+^ has also been reported in other studies [27]. However, in our study we measured a decrease in Mg^2+^ after salt stress and an increase after salt stress recovery, which is consistent with the dynamics of potassium ions (Table 1). Therefore, the Mg^2+^ in the salt tolerance of plants is likely to play a similar role to K^+^. Generally, Na^+^ uptake is limited by increased Ca^2+^ absorption, thus, ensuring ion balance. The response to salt stress in studied rice lines was accompanied by an increase in the Ca^2+^ concentration of plant tissues (Table 1). The P and microelement uptake in plants under salinity stress is complex and many other factors can also affect the absorption of P and microelements such as habitat, plant species and variety, plant growth stage, the level and composition of salt stress, and external P concentration in the growth medium [28]. This may be the reason for the differences we observed in the trends of P and microelement uptake in weedy and cultivated rice lines in this study.

### Under the salt stress, the contribution of ion transport-related genes to salt tolerance is different among four different genotypes

The present study indicates that the expression of the *HKT* and *NHX* gene families and of *SOS1* in tested rice tissues all changed over time during salt stress. Studies have found that the expression of *HKT* genes was relatively stable in shoots and higher than their expression in roots; however, their expression was higher in the roots under K^+^ starvation conditions [29]. Our findings show that the expression of *OsHKT2;1* in four different rice genotypes was not affected for up to 6 h under salt stress conditions; however, the expression was down-regulated after 24 h of stress, and the salt-tolerant population *JYGY-1* had the lowest level (Fig. 4a). As previously reported, *OsHKT2;1* mediates the influx of Na^+^ but not that of K^+^ [25]. It was observed that *OsHKT2;1* was induced by salt stress [29], and may be involved in the uptake and transport of Na^+^ from the roots to the mesophyll cells where Na^+^ causes damage.

The expression of *OsHKT2;2* was up-regulated after 6 h in the shoots in the tested genotypes in response to salt stress, with the highest expression level measured in the salt-tolerant *JYGY-1*; however, in the roots, *OsHKT2;2* was down-regulated after 6 h, most significantly in the *9311* genotype (Fig. 4b). Previous studies have found that the gene *OsHKT2;2* exists in the form of pseudogenes in *Nipponbare*, and is completely present in some salt-tolerant rice varieties [25]. In this study, the complete *OsHKT2;2* gene exists in the genotypes *9311*, *JYGY-1* and *JYFN-4* (Additional file 2: Figure S1), which is consistent with the salt tolerance level (Fig. 2). It is revealed that although *OsHKT2;2* does not mediate K^+^ uptake in severely abundant K^+^ or deficient Na^+^ conditions, it does impart salt tolerance to plants grown in high salt conditions. In *Saccharomyces cerevisiae*, it has been demonstrated that this may be achieved by potentiating K^+^ uptake [1]. Evolutionary analysis of the *HKT* transporter sequences revealed that *OsHKT2;3* and *OsHKT2;4* in rice are 93% homologous [1]. In this study, the expression of *OsHKT2;3* and *OsHKT2;4* genes in roots and shoots was significantly up-regulated under salt stress, with the largest increase in expression observed in the *JYGY-1* genotype (Fig. 4c, i). Additionally, the *OsHKT2;4* transporter has a high permeability to K^+^ relative to Na^+^ [30]. Our previous study has found that after 7 days of salt stress treatment and recovery 7d, the genotype *JYGY-1* has the lowest Na^+^/K^+^ ratio, followed by genotype J*YFN-4*, genotype *Nipponbare* is the highest (Fig. 3c). We concluded that *OsHKT2;2, OsHKT2;3,* and *OsHKT2;4* play an important role in the uptake and translocation of K^+^ by reducing the Na^+^/K^+^ ratio to improve salt tolerance in weedy rice. This is consistent with our phenotypic results and the Na^+^-K^+^ content in weedy rice and cultivated rice under salt stress.

Semi-quantitative PCR analysis showed that in 100 mM salt stress conditions, the expression of *OsHKT1;2* did not change in roots or shoots [31]. However, we found that while the expression of *OsHKT1;2* is a first down-regulation, then upregulation after the initial rise in the shoots, it is up-regulated in the roots most significantly in the *JYGY-1* genotype (Fig. 4e). This is consistent with the phenotypic observations made of rice cultivars under salt stress, indicating that *OsHKT1;2* gene expression plays an important role in improving salt tolerance in weedy rice and cultivated rice.

Electrophysiological experiments showed that in oocytes, the *OsHKT1;4* gene had the strongest selectivity for Na^+^ among cations including Li^+^, Na^+^, K^+^, Rb^+^, Cs^+^, and NH^4+^ [32]. During the reproductive stage of the *Nipponbare* line, *OsHKT1;4* concentrates Na^+^ in the leaf sheath and then excretes it from leaf blades, but the contribution of *OsHKT1;4* to sodium content is very little in the vegetative growth stage [32]. In addition, *OsHKT1;4* is expressed in all organs except young leaf sheaths and significantly decreases during the vegetative growth stage [32]. In this experiment, the rice and weedy rice were three to four leaf stage. The results showed that *OsHKT1;4* expression had decreased in the shoots and roots, indicating that the *OsHKT1;4* played a certain negative regulation in sodium ion transport process, which is consistent with the results of previous studies (Fig. 4g). Therefore, *OsHKT1;4* plays an important role in maintaining Na^+^/K^+^ balance in weedy rice and cultivated rice.

This study also found that the *OsHKT1;1*, *OsHKT1;3*, and *OsHKT1;5* genes were significantly up-regulated under salt stress after 6 h, genotype *JYGY-1* was the largest, *JYFN-4* was the second, and *Nipponbare* was the smallest.; in addition, *OsHKT1;1* expression was not affected in the roots (Fig. 4d, f, h). Studies have found that the Na^+^ content in the phloem of *OsHKT1;1* mutants was lower than that of the wild type, indicating that *OsHKT1;1* may be involved in the regulation of Na^+^ in the phloem [33]. A different study also found that the *HKT1;3* gene is highly expressed in leaf adaxial epidermal bulliform cells in response to environmental changes [34]. The rice gene *OsHKT1;5* was first identified as the quantitative trait locus *SKC1*, which encodes for a transporter that unloads Na^+^ from the root xylem [7]. The *OsSKC1* protein is a specific Na^+^ transport, and is not involved in the direct transport of K^+^. Under the salt treatment, *OsSKC1* protein could control Na^+^ concentration/transport by increasing the transport of Na^+^ from the roots to the shoots when rice plants were subjected to salt stress, alleviating Na^+^ toxicity and enhancing salt tolerance in rice [7]. This shows that during rice seedling growth, *OsHKT1;1* and *OsHKT1;5* are responsible for transporting Na^+^ to the shoot, alleviating the toxicity of the Na^+^ concentration in the roots. It can be seen that under salt stress, there is a quasi-transport of Na^+^ from not only the shoots to the roots but also from the roots to the shoots. When the concentration reaches toxic levels Na^+^ is transported to the shoots to alleviate the toxicity in the roots; however, when Na^+^ concentrations reach toxic levels in both tissues, it may cause death in the rice plants. Furthermore, in response to environmental changes, *OsHKT1;3* expression is up-regulated, which is consistent with the results from our gene expression study.

Previous studies have indicated that *NHX*-type antiporters play an important role in rice salt tolerance; *OsNHX1, OsNHX2, OsNHX3,* and *OsNHX5* can suppress Na^+^, Li^+^ accumulation in cells, and their sensitivity to high K^+^ concentrations [10]. Other studies have shown that the expression of *OsNHX1*, *OsNHX2*, *OsNHX3*, and *OsNHX5* is regulated differently in different rice plant tissues and is increased by salt stress, hyperosmotic stress, and ABA [10]. This is consistent with our findings that the *NHX* gene family members were both significantly up-regulated under salt stress in addition to the down-regulation of *OsNHX3* and *OsNHX4* in roots. The salt-tolerant *JYGY-1* rice variety had the maximum increase in *OsNHX3* and *OsNHX4* gene expression in response to salt stress (Fig. 5a-e). *OsNHX1* plays an important role in the isolation of excess cytoplasmic Na^+^ and K^+^ in vacuoles, and the amount of antiporter expressed is a meaningful index for evaluating the salt tolerance in rice [6]. Thus, the difference in *NHX* gene expression in different genotypes is one of the major factors leading to their different salt tolerance levels, and the magnitude of the difference in expression will indicate how much of a contribution each *OsNHX* gene makes to salt tolerance in that particular genotype.

The salt overly sensitive (*SOS*) pathway is involved in the expulsion of Na^+^ from the cell and maintaining an optimal cytosolic Na^+^/K^+^ ratio in the cell. The *OsSOS1* encoded Na^+^/K^+^ antiporter in plasma membrane regulates Na^+^ exclusion from the cell by transporting the ions to the apoplastic space [5]. Thus, *OsSOS1* mediated Na^+^ exclusion seems to be an important mechanism by which cells get rid of excess Na^+^. In this study, the expression of *OsSOS1* in the shoots did not change significantly after salt stress treatment, but it was significantly up-regulated in the roots (Fig. 5f). The higher expression of *OsSOS1* in both cultivated and weedy rice genotypes under salt stress would lead to the exclusion of toxic apoplastic Na^+^ from entering the internal cellular environment, and consequently leads to better salt tolerance management.

## Conclusions

Weedy rice is very close to cultivated rice in morphology and has a similar genome, but it performs better in salt stress growth conditions, which may be one of the causes of weedy rice occurrence worldwide. Due to these genetic and morphologic similarities, weedy rice provides a potential seed source for improving salt tolerance in cultivated rice varieties. Our results show that the weedy rice population *JYGY-1* has a strong salt tolerance during both seed germination and seedling growth. Weedy rice enhances its salt tolerance by regulating the Na^+^/K^+^ ratio throughout the body of the plant, as well as the absorption and transport of other ions, which is consistent with the salt tolerance mechanisms documented in rice and other plants. However, the regulation of ion homeostasis is a complex network system that requires several different genes. Together, the *OsHKT* and *OsNHX* gene families and *OsSOS1* regulate ion transport regulation under salt stress. Our results indicate that the genes related to ion transport regulation in weedy and cultivated rice share 98% homology in their cDNA and protein sequences. However, from the results of this study, the contribution of different ion transport genes in weedy rice and cultivated rice salt tolerance is different. Therefore, the results of this study will help the exploration of the key factors that lead to differences in salt tolerance between rice genotypes and will provide a reference for the cultivation of salt tolerant rice varieties.

## Methods

### Experimental material selection

We undertook an initial study of the salt tolerance of the collected 74 weedy rice populations by seed germination (with the NaCl concentration of 150, 250 and 350 mM) and seedling culture (with the NaCl concentration of 100 and 150 mM), and thus two weedy rice lines were chosen for study (data not shown). Two weedy rice lines, one *JYGY-1* (colleted from Yangzhou City, Jiangsu Province-32°59′N, 119°26′E) was salt-tolerant, whereas the other accession, *JYFN-4* (collected from Yancheng City, Jiangsu Province-33°37′N, 119°27′E) was salt-sensitive. The seed shell colors are straw without mans, and the seed peel colors are red, in addition to these, they are collected in dry direct seeding rice fields. Two rice cultivars, *Nipponbare* and *9311*, were used as comparison in this study. As the japonica rice cultivar *Nipponbare* was used for almost the whole genome sequence (International rice genome sequencing project, 2005) and approximately 28,000 full-length cDNA sequences are available [17]. *9311* is an excellent maintainer line of indica rice and one of the parents of the first super hybrid rice combination Liangyou Peijiu cultivated by two-line method in China [18]. At the same time, the genome sequence of *9311* was also published in 2002 [19].

### Seed germination

Seeds from the four different genotypes were surface sterilized with 1% (*V*/V) sodium hypochlorite solution for 30 min and washed with deionized water three times. Unless otherwise stated, 20 seeds were placed evenly in each Petri dish (9 cm in diameter) lined with two layers of filter paper moistened with 5 ml deionized water (pH 6.6) containing 0, 150, 200, 250, 300, 350, and 400 mM NaCl. Four replicates for each experiment were placed in different petri dishes. Each Petri dish was sealed with plastic paraffin film and placed in an incubator with a 12 h light/12 h dark photoperiod regime at 30 °C /25 °C. The light intensity was 140 μmol m^− 2^ s^− 1^ photosynthetic photon flux density (PPFD) provided by fluorescent lamps, and the growth chamber was maintained at 60% relative humidity. The salt stress treatment duration was two weeks. Seeds were considered germinated when the radicle was protruding from the seed coat. The number of germinated seeds was counted each week.

### Seedling culture and salt stress treatment

Seeds imbibed in deionized water for 24 h at 25 °C and were germinated in Petri dishes with two layers of wet filter paper at 30 °C in dark for 3 d. The uniformly germinated seeds were selected and cultivated in a plastic pot (10.3 cm × 9 cm × 5.4 cm) filled with IRRI (International Rice Research Institute) nutrient solution [20], and 20 plants were maintained in each pot. Four replicates for each experiment were placed in different plastic pots. The seedlings were grown at 25 °C/20 °C and were maintained photoperiodic cycle of 14 h light and 10 h dark with light intensity of mentioned above. When seedlings had three fully expanded leaves (about 2 weeks after sowing), they were treated with nutrient solution containing 150 mM NaCl for 1 week, and followed by recovery with IRRI nutrient solution for 1 week, and then to measure their survival rate. In the 150 mM salt concentration, the sensitive plants were dead and dried-up and the results of the phenotypic experiments were clearly showed. However, the results of the determination of the ion content and the expression of genes were greatly affected. Therefore, 125 mM salt concentration was used for measure ion content and the expression of genes. In addition to that the seedings were treated with 125 mM NaCl after 0, 6, 24 and 72 h were harvested, and immediately frozen with liquid nitrogen, and stored at − 80 °C, and were prepared for the quantitative real-time PCR experiments.

### Determination of Na^+^ and K^+^ content

Shoots and roots of weedy and cultivated rice seedlings were sampled separately and the concentration of copper (Cu^2+^), manganese (Mn^2+^), iron (Fe^2+^), zinc (Zn^2+^), calcium (Ca^2+^), magnesium (Mg^2+^), potassium (K^+^), phosphorus (P), and sodium (Na^+^) was measured according to the method provided by Ali et al. [21], with minor modification. Briefly, samples were dried in 80 °C for 5 d. Each dried sample was boiled for 8 h at 90 °C in 5 ml of nitric acid guaranteed reagent (GR), dilute with distilled water to a final volume of 25 ml, and analyzed using an inductively coupled plasma-optical emission spectrometry instrument (ICP-OES; Pekin Elmer, Norwalk, CT, USA). Finally calculate the Na^+^/K^+^ ratio according to the ion concentrations.

### Gene identification by Illumina sequencing and homology comparison

Relevant gene sequences were obtained by Illumina sequencing using methods described by Xu et al., with minor modification. A brief description is total RNA was extracted from 5 to 6 entire weedy rice plants using RNAiso Plus (TaKaRa Biotech, Japan) according to the instructions for use of the product. The concentration and the quality of RNA were analyzed using a Nanodrop ND1000 (Nanodrop Technologies, Wilmington, USA) and Ultrasec™ 2100 pro U*V*/Visible spectrophotometer (Amersham Biosciences, Uppsala, Sweden). Equal quantities of total RNA from three independent samples were used for RNA-Seq and we used BlastN (version 2.2.23) alignment against known ion transport-related genes in rice. The rice gene sequences (*OsHKT*, *OsNHX*, and *OsSOS1*) were obtained from NCBI (https://www.ncbi.nlm.nih.gov/) and RAP-DB (http://rapdb.dna.affrc.go.jp/). The sequences were analyzed and compared using BioEdit (Ibis Biosciences Co., Ltd. CA, USA) Sequence Alignment Editor Software.

### Quantitative real-time PCR

Twenty plants of each genotype were grown to the three- to four-leaf stage under the cultivated conditions described above. The plants of the above ground part were used to extract RNA after 0, 6, 24 and 72 h with salt treated (125 mM). Total RNA was extracted from each plant and the quality and quantity of total RNA were analyzed according to the method described above. Total RNA was reverse-transcribed using PrimeScript™ RT reagent kit with gDNA Eraser (TaKaRa Biotech) according to the instructions for use of the product. The amplification of *actin1* (accession no. AB047313) was used as internal control genes, since it is considered as relatively stable genes in rice [22]. All of the genes related to ionic homeostasis and internal control gene were used to design primers for quantitative real-time PCR (qRT-PCR) (Table 2). The Quantitative real-time PCR (qRT-PCR) method with reference to Pan et al. [23].

**Table 2:** Primer list for the qRT-PCR analysis of rice and weedy rice genes

Accession	Gene	Forward primer (5′-3′)	Reverse primer (5′-3′)	Fragment size (bp)
AB061311	OsHKT2;1	TCGGCAAGCACTGTGATAAG	AGCGACATGGATCACAACAA	156
AB061313	*OsHKT2;2*	GCTTCCTAAGTTGCCGACTG	TGAGTGAGCAGTCGATGGAG	197
AJ491820	*OsHKT2;3*	GCTGGTCTGCATCACTGAAA	GCAGAGCATTGCAAGAACAA	235
AJ491816	*OsHKT1;1*	CCTTTTGCATCTTCACAGCA	ATACGCATAGCCGCAAGAGT	165
KT795742	*OsHKT1;2*	ATCCACGTCGTTCTTCTCGT	GTCCTCTTCACCCGGTTCTT	192
AJ491818	*OsHKT1;3*	GGATTTCTCAAGCGCTCAAC	CCATGCGGAGTTCAGAAAAT	233
AK109852	*OsHKT1;4*	CATCTGCATCACCGAGAGAA	CTCCCTACGAAACCAGTCCA	180
AK108663	*OsHKT1;5; SKC1*	CCCATCAACTACAGCGTCCT	AACTTCTTGAGCCTGCCGTA	206
AJ491855	*OsHKT2;4*	CTTGGTTTTGTTGCCTTGGT	GGCCAAGAAAGGAAAGGAAC	205
AB021878	*OsNHX1*	GCTAGATTTGAGCGGCATTC	CACTGGCAAACTCCCATTTT	197
AB531435	*OsNHX2*	TGGATCAAGGAAGGATTTCG	AAGGTCAGGCCACACTCAAC	193
AB531433	*OsNHX3*	ATGGATGCACTGGACATTGA	TCTTTGGGCGTGTCTCTTTT	166
AP003507	*OsNHX4*	CGCCAATCACATTCAATCAC	GCCTTGTCAAGAAGCCAAAC	192
AB531434	*OsNHX5*	CTTCCTGGAGGACATGGAAA	AACGATGTCGTGCTTTTGTG	248
AY785147	*OsSOS1*	TAAGCAGCAGGCATTCATTG	AAAGCCTGGCAACGACTAGA	206
AB047313	*Actin1*	CTGCGGGTATCCATGAGACT	TGGAATGTGCTGAGAGATGC	247

### Data analyses

The experiments were designed with random complete block and repeated three times. Each replication was considered as a block and arranged in different Petri dishes or pots in the incubators. All experiments were performed twice. The experimental results were tested by one-way analysis of variance. Data were incorporated for analysis based on each experiment without significant (α > 0.05) trial-by-treatment interaction. Unless otherwise noted, the data are means ± standard error (SE). Data was fitted to a four-parameter non-linear logistic-regression model using SigmaPlot ver. 10.0 (Systat Software, Inc., CA, USA). Differences among the treatment means were evaluated using Duncan’s Multiple Range test at *P* < 0.05. Data analyses were conducted using SPSS ver. 21.0 (IBM, USA).

## Additional files

Additional file 1: **Table S1.** Gene sequences were obtained by Illumina sequencing and homology studies between cultivated and weedy rice genotypes. (DOCX 19 kb)

Additional file 2: **Figure S1.** Comparison of amino acid change sites of ion transport related genes (*HKT* and *NHX* gene families and *SOS1*) between weedy rice genotype *JYGY-1* and cultivated rice genotype *Nipponbar*. (W) stands for weedy rice genotype *JYGY-1*, and (R) stands for cultivated rice genotype *Nipponbar* (Except for the gene *OsHKT2;2*). Among them, the gene with the most amino acid change between weedy rice and rice is *OsNHX4*, as there are eight amino acid changes identified, followed by *OsHKT2;2*, *OsHKT2;3*, *OsHKT2;4*, *OsHKT1;5*, Os*NHX2*, *OsHKT1;1*, Os*SOS1* and *OsHKT1;4.* And the homology of other genes not shown is 100%. (PDF 2709 kb)

## Abbreviations

GR: Guaranteed reagent
*HKT*: High-affinity K+ transporter
ICP-OES: Inductively coupled plasma-optical emission spectrometry instrument
IRRI: International Rice Research Institute
*NHX*: Tonoplast sodium-hydrogen exchanger
PPFD: Photosynthetic photon flux density
QRT-PCR: Quantitative real-time PCR
*SOS1*: Salt overly sensitive 1
