# Supplementary material for: Na+/K+ Balance and Transport Regulatory Mechanisms in Weedy and Cultivated Rice (Oryza sativa L.) Under Salt Stress
Source: BMC Plant Biol. 2018 Dec 29;18:375. doi: 10.1186/s12870-018-1586-9 (PMC6311050; doi:10.1186/s12870-018-1586-9)
Supplement: Supplementary file 1 — Table S1. Gene sequences were obtained by Illumina sequencing and homology studies between cultivated and weedy rice genotypes. (DOCX 19 kb) [file 12870_2018_1586_MOESM1_ESM.docx]

**Table S1.** Gene sequences were obtained by Illumina sequencing and homology studies between cultivated and weedy rice genotypes

| Rice | |  | Weedy Rice | | | |  | Sequence Homology (%) | |
| --- | --- | --- | --- | --- | --- | --- | --- | --- | --- |
| Accession | Gene |  | Accession* | Size (bp) | E-value (E)** | Score (S)*** |  | cDNA | Amino acid |
| AB061311 | *OsHKT2;1* |  | KY752536 | 1976 | 0 | 2476 |  | 99.75 | 100 |
| AB061313 | OsHKT2;2 |  | KY752537 | 1975 | 0 | 3087 |  | 98.93 | 98.30 |
| AJ491820 | OsHKT2;3 |  | KY752538 | 1628 | 0 | 1957 |  | 99.15 | 98.43 |
| AJ491816 | OsHKT1;1 |  | KY752539 | 2046 | 0 | 3241 |  | 99.70 | 99.46 |
| KT795742 | OsHKT1;2 |  | KY752540 | 1238 | 5.00E^-123^ | 1189 |  | 100.00 | 100 |
| AJ491818 | OsHKT1;3 |  | KY752540 | 2091 | 0 | 2027 |  | 100.00 | 100 |
| AK109852 | OsHKT1;4 |  | KY752542 | 1280 | 0 | 1302 |  | 99.80 | 99.80 |
| AK108663 | OsHKT1;5; OsSKC1 |  | KY752543 | 2072 | 0 | 1396 |  | 99.58 | 99.10 |
| AJ491855 | OsHKT2;4 |  | KY752544 | 1633 | 0 | 1877 |  | 99.54 | 98.82 |
| AB021878 | Os*NHX1* |  | KY752545 | 2227 | 0 | 3188 |  | 100 | 100 |
| AB531435 | Os*NHX2* |  | KY752546 | 1981 | 0 | 3017 |  | 99.15 | 99.27 |
| AB531433 | Os*NHX3* |  | KY752547 | 1921 | 0 | 1465 |  | 100 | 100 |
| AP003507 | Os*NHX4* |  | KY752548 | 3276 | 1.00E^-178^ | 1304 |  | 99.29 | 98.93 |
| AB531434 | Os*NHX5* |  | KY752549 | 2317 | 0 | 3172 |  | 99.99 | 100 |
| AY785147 | Os*SOS1* |  | KY752550 | 3816 | 0 | 6786 |  | 99.99 | 99.74 |

* The accession is obtained from NCBI (<https://www.ncbi.nlm.nih.gov/>) where weedy rice gene sequences (*HKT*, *NHX*, and *SOS1*) have been uploaded.

**The E value is an evaluation of the reliability of the S value. An E value of less than 10^-6^ indicates very high homology between genes.

***The S value indicates the homology of the two sequences, and a higher value indicates a greater degree of similarity.
