# Supplementary material for: Na+/K+ Balance and Transport Regulatory Mechanisms in Weedy and Cultivated Rice (Oryza sativa L.) Under Salt Stress
Source: BMC Plant Biol. 2018 Dec 29;18:375. doi: 10.1186/s12870-018-1586-9 (PMC6311050; doi:10.1186/s12870-018-1586-9)
Supplement: Supplementary file 2 — Figure S1. Comparison of amino acid change sites of ion transport related genes (HKT and NHX gene families and SOS1) between weedy rice genotype JYGY-1 and cultivated rice genotype Nipponbar. (W) stands for weedy rice genotype JYGY-1, and (R) stands for cultivated rice genotype Nipponbar (Except for the gene OsHKT2;2). Among them, the gene with the most amino acid change between weedy rice and rice is OsNHX4, as there are eight amino acid changes identified, followed by OsHKT2;2, OsHKT2;3, OsHKT2;4, OsHKT1;5, OsNHX2, OsHKT1;1, OsSOS1 and OsHKT1;4. And the homology of other genes not shown is 100%. (PDF 2709 kb) [file 12870_2018_1586_MOESM2_ESM.pdf]

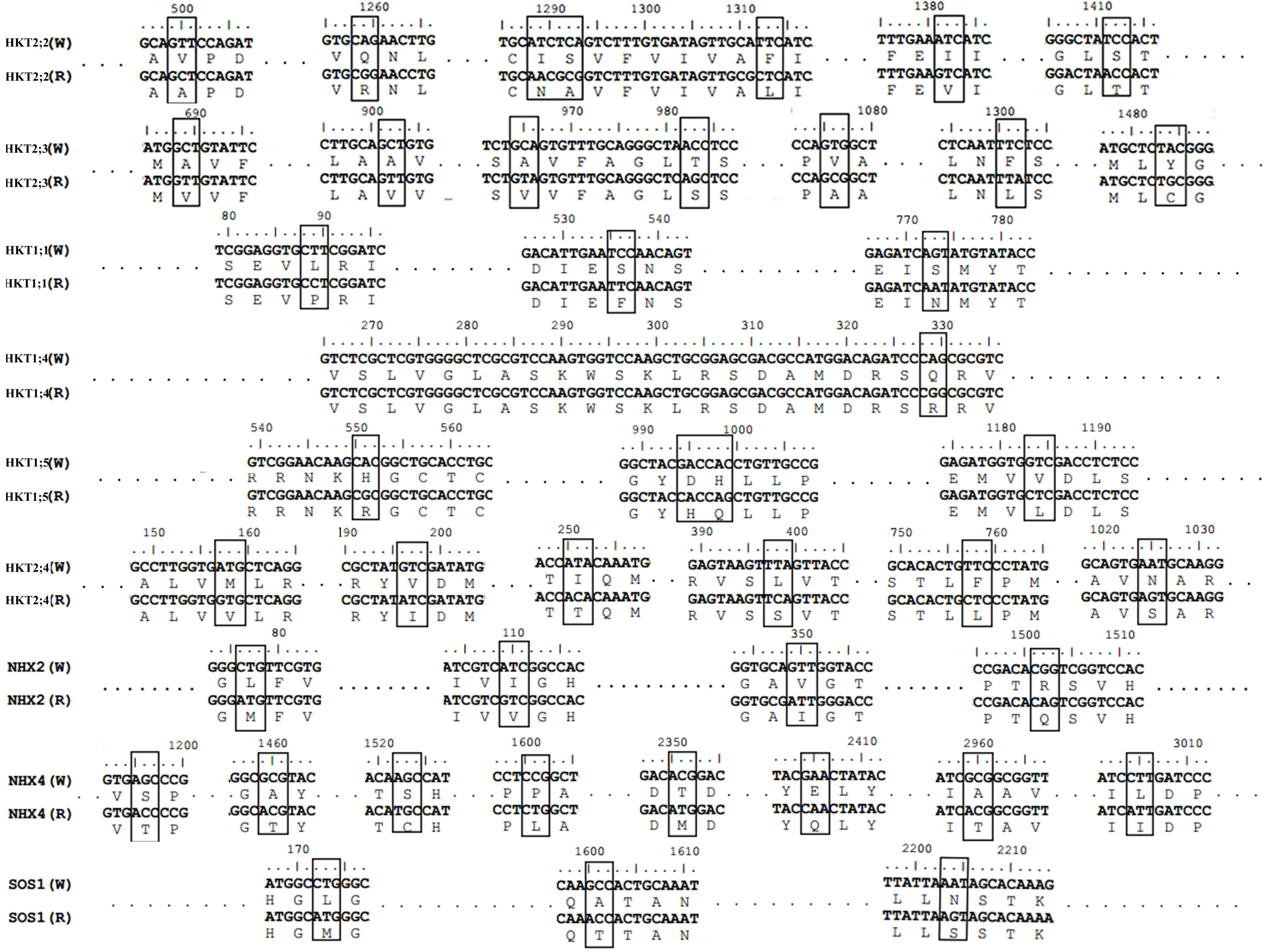
HKT2;2(W)
HKT2;2(R)
500
GCAGTTCCAGAT
A V P D
GCAGCTCCAGAT
A A P D
1260
GTGCAGAACTTG
V Q N L
GTGCGGAACCTG
V R N L
1290 1300 1310
TGCATCTCAGTCTTTGTGATAGTTGCATTCATC
C I S V F V I V A F I
TGCAACGCGGTCTTTGTGATAGTTGCGCTCATC
C N A V F V I V A L I
1380
TTTGAAATCATC
F E I I
TTTGAAGTCATC
F E V I
1410
GGGCTATCCACT
G L S T
GGACTAACCACT
G L T T
HKT2;3(W)
HKT2;3(R)
690
ATGGCTGTATTC
M A V F
ATGGTTGTATTC
M V V F
900
CTTGCAGCTGTG
L A A V
CTTGCAGTTGTG
L A V V
970 980
TCTGCAGTGTTTGCAGGGCTAACCTCC
S A V F A G L T S
TCTGTAGTGTTTGCAGGGCTCAGCTCC
S V V F A G L S S
1080
CCAGTGGCT
P V A
CCAGCGGCT
P A A
1300
CTCAATTTCTCC
L N F S
CTCAATTTATCC
L N L S
1480
ATGCTCTACGGG
M L Y G
ATGCTCTGCGGG
M L C G
HKT1;1(W)
HKT1;1(R)
80 90
TCGGAGGTGCTTCGGATC
S E V L R I
TCGGAGGTGCCTCGGATC
S E V P R I
530 540
GACATTGAATCCAACAGT
D I E S N S
GACATTGAATTCAACAGT
D I E F N S
770 780
GAGATCAGTATGTATACC
E I S M Y T
GAGATCAATATGTATACC
E I N M Y T
HKT1;4(W)
HKT1;4(R)
270 280 290 300 310 320 330
GTCTCGCTCGTGGGGCTCGCGTCCAAGTGGTCCAAGCTGCGGAGCGACGCCATGGACAGATCCCAGCGCGTC
V S L V G L A S K W S K L R S D A M D R S Q R V
GTCTCGCTCGTGGGGCTCGCGTCCAAGTGGTCCAAGCTGCGGAGCGACGCCATGGACAGATCCCGGCGCGTC
V S L V G L A S K W S K L R S D A M D R S R R V
HKT1;5(W)
HKT1;5(R)
540 550 560
GTCGGAACAAGCACGGCTGCACCTGC
R R N K H G C T C
GTCGGAACAAGCGCGGCTGCACCTGC
R R N K R G C T C
990 1000
GGCTACGACCACCTGTTGCCG
G Y D H L L P
GGCTACCACCAGCTGTTGCCG
G Y H Q L L P
1180 1190
GAGATGGTGGTCGACCTCTCC
E M V V D L S
GAGATGGTGCTCGACCTCTCC
E M V L D L S
HKT2;4(W)
HKT2;4(R)
150 160
GCCTTGGTGATGCTCAGG
A L V M L R
GCCTTGGTGGTGCTCAGG
A L V V L R
190 200
CGCTATGTCGATATG
R Y V D M
CGCTATATCGATATG
R Y I D M
250
ACCATACAAATG
T I Q M
ACCACACAAATG
T T Q M
390 400
GAGTAAGTTTAGTTACC
R V S L V T
GAGTAAGTTCAGTTACC
R V S S V T
750 760
GCACACTGTTCCCTATG
S T L F P M
GCACACTGCTCCCTATG
S T L L P M
1020 1030
GCAGTGAATGCAAGG
A V N A R
GCAGTGAGTGCAAGG
A V S A R
NHX2 (W)
NHX2 (R)
80
GGGCTGTTCGTG
G L F V
GGGATGTTCGTG
G M F V
110
ATCGTCATCGGCCAC
I V I G H
ATCGTCGTCGGCCAC
I V V G H
350
GGTGCAGTTGGTACC
G A V G T
GGTGCGATTGGGACC
G A I G T
1500 1510
CCGACACGGTCGGTCCAC
P T R S V H
CCGACACAGTCGGTCCAC
P T Q S V H
NHX4 (W)
NHX4 (R)
1200
GTGAGCCCG
V S P
GTGACCCCG
V T P
1460
GGCGCGTAC
G A Y
GGCACGTAC
G T Y
1520
ACAAGCCAT
T S H
ACATGCCAT
T C H
1600
CCTCCGGCT
P P A
CCTCTGGCT
P L A
2350
GACACGGAC
D T D
GACATGGAC
D M D
2410
TACGAACTATAC
Y E L Y
TACCAACTATAC
Y Q L Y
2960
ATCGCGGCGGTT
I A A V
ATCACGGCGGTT
I T A V
3010
ATCCTTGATCCC
I L D P
ATCATTGATCCC
I I D P
SOS1 (W)
SOS1 (R)
170
ATGGCCTGGGC
H G L G
ATGGCATGGGC
H G M G
1600 1610
CAAGCCACTGCAAAT
Q A T A N
CAAACCACTGCAAAT
Q T T A N
2200 2210
TTATTAAATAGCACAAAG
L L N S T K
TTATTAAGTAGCACAAAA
L L S S T K
